# Some Remarks on Hernia

**Published:** 1908-10-31

**Authors:** 


### Surgery.

### SOME REMARKS ON HERNIA.

### IV.—IRREDUCIBILITY.

A hernia may be completely or partially irreducible from the date of its appearance, or it may become so subsequently. Irreducibility is often the first sign that a hernia is becoming strangulated. Every strangulated hernia is irreducible, but it does not follow that every irreducible hernia is strangulated. The causes of irreducibility are manifold. Thus when this sign makes its appearance in a hernia of many years' standing, it may be due to mere bulk. The abdominal cavity may have become accustomed to the altered state of affairs, and can now no longer accommodate the extruded coils in spite of all attempts to replace them; or, again, changes may occur in the coils of intestine in the sac itself, which prevent reduction; a deposit of fat in the appendices epiploicæ, if there is any colon in the hernia, or an overgrowth of adipose tissue in the omentum in an epiplocele may produce this result. But the commonest cause of simple irreducibility is the formation of adhesions between the constituents of the hernia and the sac wall. It is generally the omentum which becomes adherent in this way; adhesions between the sac and the intestine itself are not common. More rarely adhesions occur between different parts of the sac itself leading to the formation of bands, which prevent reduction. Such adhesions are nearly always found in herniæ which have been subjected to truss pressure for any length of time.

A hernia may become irreducible without any serious symptoms supervening, and some patients suffer no inconvenience, while others complain of vague colicky pains and dragging sensations referred to the site of attachment of the mesentery. It is not in itself a condition necessarily demanding operation. Much may be done by keeping the patient in a supine position on an antifat diet, in the hope that the hernia may diminish in size by these means, and then reduce spontaneously. But it is never possible to say with certainty whether the irreducibility is due to increase in the bulk of the hernia only, or to the presence of adhesions. In the latter case the treatment will, of course, be unsuccessful. Further it must be remembered that a patient with an irreducible hernia runs a greater risk of strangulation than the possessor of an ordinary hernia; and it is not possible to apply pressure to such a hernia with a truss. There is then every argument in favour of operating, and performing a radical cure. It might, however, be argued that where the irreducibility is due to bulk, it would be found impossible at the operation to return the contents of the hernia to the abdomen. But the increase in bulk is nearly always due to omentum, and this can be ligatured and removed without causing the patient any serious inconvenience. As a general rule, therefore, and supposing that no general contra-indications exist, a radical cure should be advised in all cases of irreducible hernia, whether that irreducibility is primary, or comes on later.

So far, simple irreducibility has been discussed; that is to say the irreducibility is the only abnormality. But it may be merely a symptom of graver trouble. The following varieties of hernia are all irreducible: (*a*) Inflamed; (*b*) obstructed; (*c*) strangulated. An inflamed hernia is one in which inflammatory changes have occurred in the sac. The common causes are the pressure of an ill-fitting truss, or a blow. The hernia becomes irreducible, painful and tender. The skin covering it is reddened. An impulse can still be obtained when the patient coughs. There is not usually any vomiting, and the bowels, though constipated, will react to the ordinary methods of purgation. The temperature is raised. Literally speaking, the title would also include all varieties of inflammation occurring in the contents of a hernia leading to changes in the sac. Thus an appendix has been known to descend into the sac of a hernia, and become inflamed, causing appendicitis in a hernial sac.

Some authorities advocate palliative treatment and the application of an icebag to the part; but on the whole it is a simpler, and certainly a safer, plan to operate at once. It is impossible to predict the exact state of affairs which will be found when the sac is opened; and it is better therefore to operate in any case. If the inflammation is acute, the wound should be drained, and a radical cure may be undertaken subsequently when the inflammation has subsided.

An obstructed hernia is, as has been said, also irreducible. It is one in which there is obstruction to the passage of the contents of the extruded coils only. It is therefore essentially different to a strangulated hernia, but it may be a precursor of this condition. Such a hernia nearly always contains colon; this is only what would be expected when one remembers that the contents of the small intestine are liquid, except possibly in the very lowest portion of the ileum. Constipation is an almost constant factor in its causation. The symptoms which it causes are vague at first. The rupture becomes larger and tenser, and, if it was reducible before, it is now irreducible. Anorexia, constipation and colicky pains are complained of. Vomiting occurs later. When the hernia is examined, it will be found to be tense and tender, and above all it imparts a doughy sensation to the hand, due to the retained fæcal matter in the contained intestine. An impulse can nearly always be obtained at the neck of the sac, but it is generally a faint one. This condition may subside spontaneously, owing to the passage of the fæcal matter, or it may, if left, go on to strangulation. An enema should be given to clear the large intestine, and start peristalsis. An opium fomentation should then be applied, and this often produces sufficient relaxation of the part, to enable the hernia to be reduced by gentle taxis. If, however, it is still irreducible, operation should not be delayed. Under no circumstances should a purge be given by the mouth.

